# Tumor-Associated Endothelial Cells Promote Tumor Metastasis by Chaperoning Circulating Tumor Cells and Protecting Them from Anoikis

**DOI:** 10.1371/journal.pone.0141602

**Published:** 2015-10-28

**Authors:** Arti Yadav, Bhavna Kumar, Jun-Ge Yu, Matthew Old, Theodoros N. Teknos, Pawan Kumar

**Affiliations:** 1 The Ohio State University Comprehensive Cancer Center, Columbus, Ohio, 43210, United States of America; 2 Department of Otolaryngology-Head and Neck Surgery, The Ohio State University, Columbus, Ohio, 43210, United States of America; INSERM, FRANCE

## Abstract

Tumor metastasis is a highly inefficient biological process as millions of tumor cells are released in circulation each day and only a few of them are able to successfully form distal metastatic nodules. This could be due to the fact that most of the epithelial origin cancer cells are anchorage-dependent and undergo rapid anoikis in harsh circulating conditions. A number of studies have shown that in addition to tumor cells, activated endothelial cells are also released into the blood circulation from the primary tumors. However, the precise role of these activated circulating endothelial cells (CECs) in tumor metastasis process is not known. Therefore, we performed a series of experiments to examine if CECs promoted tumor metastasis by chaperoning the tumor cells to distal sites. Our results demonstrate that blood samples from head and neck cancer patients contain significantly higher Bcl-2-positive CECs as compared to healthy volunteers. Technically, it is challenging to know the origin of CECs in patient blood samples, therefore we used an orthotopic SCID mouse model and co-implanted GFP-labeled endothelial cells along with tumor cells. Our results suggest that activated CECs (Bcl-2-positive) were released from primary tumors and they co-migrated with tumor cells to distal sites. Bcl-2 overexpression in endothelial cells (EC-Bcl-2) significantly enhanced adhesion molecule expression and tumor cell binding that was predominantly mediated by E-selectin. In addition, tumor cells bound to EC-Bcl-2 showed a significantly higher anoikis resistance via the activation of Src-FAK pathway. In our *in vivo* experiments, we observed significantly higher lung metastasis when tumor cells were co-injected with EC-Bcl-2 as compared to EC-VC. E-selectin knockdown in EC-Bcl-2 cells or FAK/FUT3 knockdown in tumor cells significantly reversed EC-Bcl-2-mediated tumor metastasis. Taken together, our results suggest a novel role for CECs in protecting the tumor cells in circulation and chaperoning them to distal sites.

## Introduction

Head and neck squamous cell carcinoma (HNSCC) is the 8th most frequent cancer worldwide and five-year survival rates (<50%) are among the lowest of the major cancers [1, 2]. Although advancements in the anti-cancer treatments including surgery, radiation and chemotherapy have increased the local control of HNSCC, the overall survival rates have not improved significantly over the last three decades [3, 4]. Five year survival rates for patients with early stage localized head and neck cancers are more that 80% but drops to 40% when the disease has spread to the regional neck nodes, and to below 20% for patients with distant metastatic disease [3]. A number of studies have highlighted the role of tumor microenvironment in promoting tumor metastasis [5–7]. We have previously demonstrated that VEGF, in addition to its pro-angiogenic function, also induces the expression of Bcl-2 in the microvascular endothelial cells [8]. We have recently shown that tumor-associated endothelial cells exhibit significantly higher Bcl-2 expression that is directly correlated with metastatic status of head and neck cancer patients [6, 9]. In addition, overexpression of Bcl-2 alone in tumor-associated endothelial cells was sufficient to promote tumor metastasis in a SCID mouse model [6].

Metastatic process is highly complex and it involves multiple steps including the release of tumor cells from the primary tumor, survival in circulation, interaction with vascular endothelium and invasion of target organs [10]. Although millions of tumor cells are released in circulation each day, only a few of these tumor cells are able to successfully complete the metastatic journey [11]. This could be due to the fact that most of the cancer cells, particularly epithelial cells require adhesion to other cells or extracellular matrix (ECM) to survive and proliferate [12–14]. When epithelial cells lose their normal cell-matrix interactions, the cell cycle is arrested and cell undergoes a rapid caspase-mediated cell death, known as anoikis [15]. In adherent cells, cell-specific activation of integrins and their downstream signaling mediators promote cell survival through interactions with cytoplasmic kinases, small G-proteins and scaffolding proteins [16–18]. Integrin ligation activates FAK, a nonreceptor tyrosine kinase, and activated FAK phosphorylates itself and other cellular proteins [16]. FAK autophosphorylation at Y397 provides a binding site for SH2 domain-containing proteins such as Src family kinases and PI3K subunit p85 [19, 20]. Activation of these signaling pathways plays a central role in anoikis resistance. In addition to circulating tumor cells, increased levels of viable circulating endothelial cells are also observed in cancer patients with progressive disease [21]. Mancuso and co-workers [22] have also shown increased levels of activated endothelial cells in cancer patients. Results obtained from this study also demonstrate that blood samples from head and neck cancer patients contain significantly higher Bcl-2 positive (activated) circulating endothelial cells as compared to healthy volunteers.

In this study, we investigated if circulating endothelial cells could provide a temporary substratum to the circulating tumor cells (CTCs) to protect them from anoikis and chaperone these CTCs to distal sites. Our results show that endothelial cells overexpressing Bcl-2 (EC-Bcl-2) expressed significantly higher levels of E-selectin and exhibited enhanced tumor cell binding. In addition, tumor cells bound to EC-Bcl-2 showed significantly higher anoikis resistance that was mediated by the Src-FAK signaling pathway. Furthermore, SCID mice co-injected with tumor cells and EC-Bcl-2 showed significantly higher lung metastasis, that was markedly reversed by the knockdown of E-selectin in EC-Bcl-2 and FAK or FUT3 knockdown in tumor cells. Taken together our results demonstrate a novel role for tumor-associated endothelial cells in protecting tumor cells from anoikis and chaperoning the tumor cells to distal sites.

## Results

### Blood samples from head and neck cancer patients showed significantly higher circulating Bcl-2 positive endothelial cells

**Fig 1:**
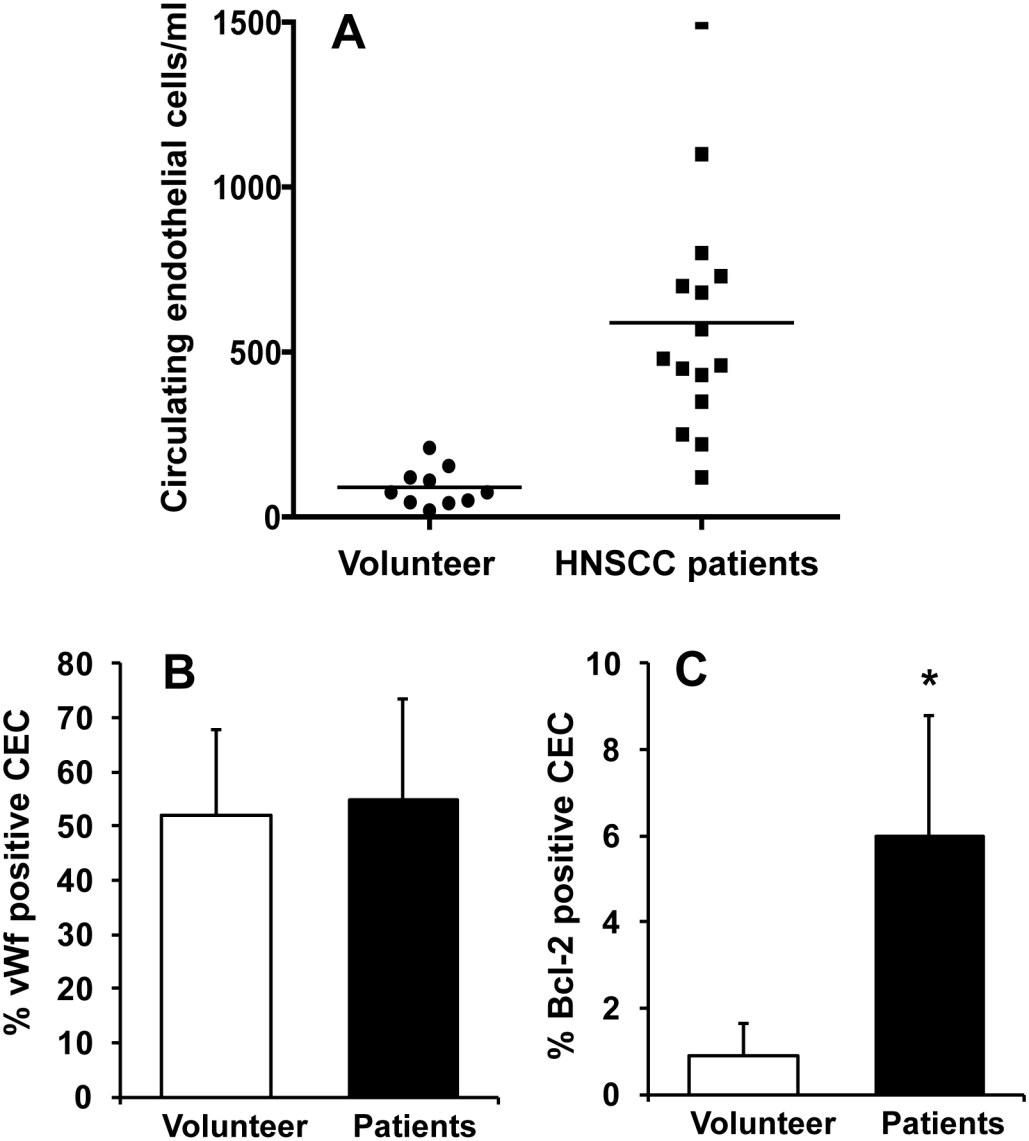
Bcl-2 positive circulating endothelial cell (CEC) levels are higher in cancer patients. CECs were isolated from the blood samples of head and neck cancer patients (n = 15) and healthy volunteers (n = 10) using anti-CD146 antibody and Easy Step magnetic nanoparticles (Stem Cell Technology). **A;** The purified CECs were counted and statistical significance was analyzed by Mann-Whitney test (p value <0.0001). CECs were then cytospun on glass slides and stained for factor VIII (vWf, **B**) or Bcl-2 (**C**). *, represent a significant difference (p<0.01) in Bcl-2 positive CECs in head and neck cancer patients as compared to healthy volunteers.

We examined circulating endothelial cell (CEC) levels in 25 blood samples (15 head and neck cancer patients and 10 healthy volunteers). Our results show that blood samples from head and neck cancer patients contained significantly higher CECs as compared to blood samples from healthy volunteers (267 vs 57; p<0.0001, Mann-Whitney test, Fig 1A). We have previously demonstrated that tumor samples from head and neck cancer patients have significantly higher Bcl-2 positive tumor vessels as compared to matched normal controls [6, 9]. In this study, we further examined if blood samples from head and neck cancer patients also contained elevated levels of Bcl-2 positive CECs. We stained the CECs for Von Willibrand Factor (Factor VIII, an endothelial cell marker) and Bcl-2. A similar level of factor VIII staining was observed in CECs from head and neck cancer patients and healthy volunteers (Fig 1B). In contrast, CECs from head and neck cancer patients showed marked increase in Bcl-2 levels as compared to CECs from healthy volunteers (Fig 1C).

### Tumor-associated endothelial cells and circulating tumor cells are present in blood and co-migrate to lungs of tumor bearing mice

**Fig 2:**
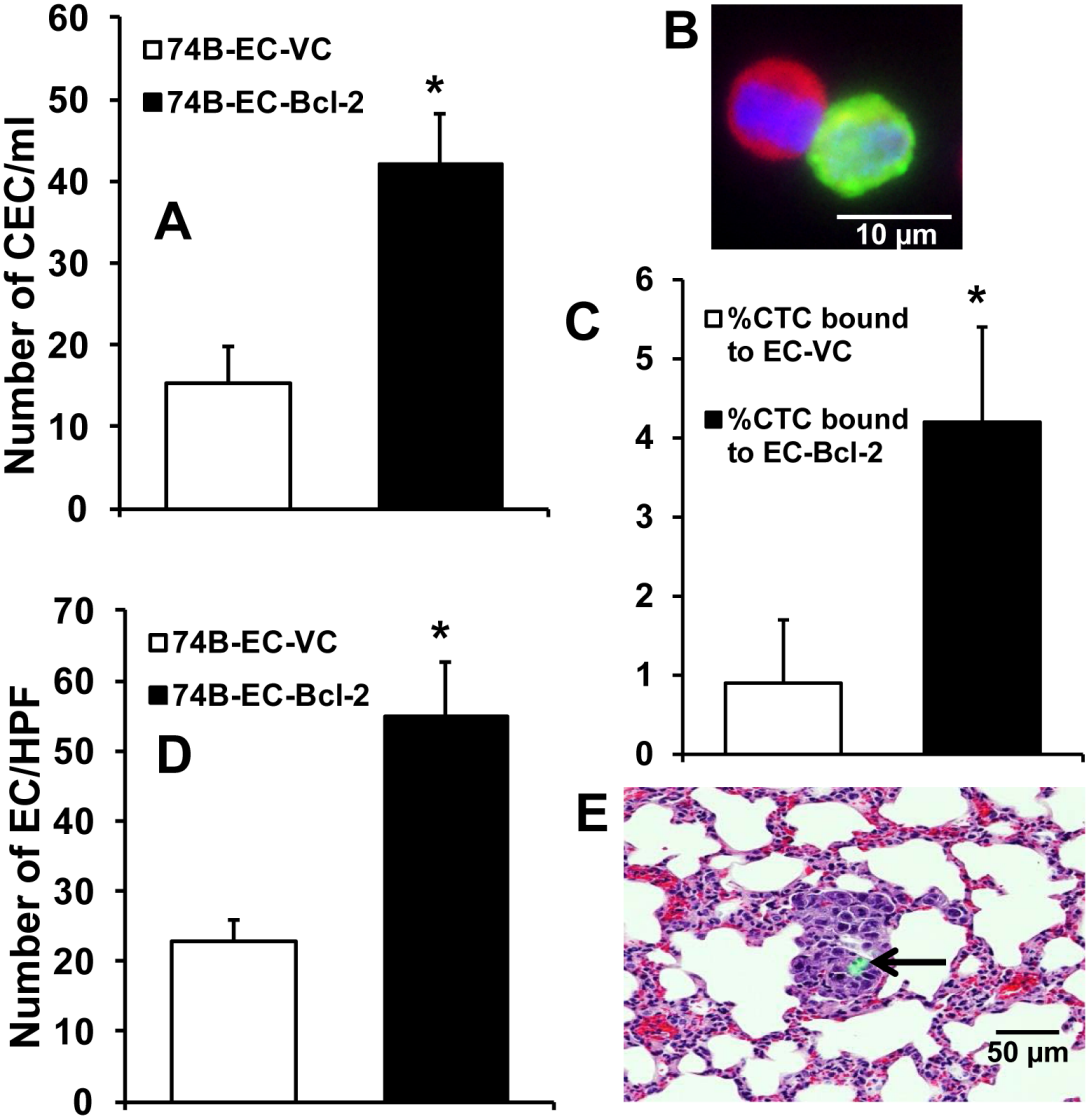
Bcl-2 positive tumor-associated endothelial cells co-migrate to lungs. Green fluorescent protein (GFP)-labeled EC-Bcl-2 or EC-VC were co-implanted with tumor cells at the floor of the mouth. After 14 days, blood samples and lungs were collected. **A-B;** Circulating endothelial cells (CECs, GFP-labeled) were purified by negative selection method. **A;** GFP-labeled CECs were counted and *, represent a significant difference (p<0.01) in CEC numbers in animals bearing tumor cells and EC-Bcl-2 as compared to animals bearing tumor cells and EC-VC. **B;** A representative picture showing CTC (red) bound to CEC (green). Separate pictures for GFP-EC-Bcl-2 (green) and CTC (cytokeratin staining, red) were taken by Nikon Eclipse 80i microscope with DS-Ril camera and overlaid using NIS-Elements-Basic Research software (Nikon). The scale bar represents 10 μm. C; Circulating tumor cells (CTCs) bound to EC-VC or EC-Bcl-2 were counted and expressed as %CTC bound. **D-E;** Lung sections were analyzed by IHC for co-migration of EC-GFP and tumor cells. **D;** Number of GFP-CECs present in lung sections were counted. **E;** A representative picture showing GFP-labeled ECs (black arrow) along with tumor nodules in lung. The scale bar represents 50 μm.

Our CEC results suggest that Bcl-2 positive tumor-associated endothelial cells may be released from the primary tumors in circulation. Technically, it is quite challenging to pin-point the origin of these Bcl-2 positive CECs in cancer patients. Therefore, we used a SCID mouse model to investigate if tumor- associated endothelial cells are released in the circulation and migrate to lungs. We labeled EC-Bcl-2 and EC-VC with GFP. These GFP positive endothelial cells were co-implanted with tumor cells in the floor of the mouth of SCID mice and the release of these GFP labeled endothelial cells and migration to lungs was tracked by collecting blood samples and lungs at day 14. Blood samples from mice bearing tumors with GFP-labeled EC-Bcl-2 showed significantly higher CECs as compared to mice bearing tumors with GFP-labeled EC-VC (Fig 2A). In addition, significantly higher CTCs were found to be bound to EC-Bcl-2 as compared to EC-VC (Fig 2C). Fig 2B shows the presence of circulating tumor cells (CTCs, red) bound to GFP-labeled human endothelial cells (green) in the blood samples from mice bearing tumors with GFP-labeled EC-Bcl-2. Similarly, a significantly higher number of GFP positive EC-Bcl-2 cells were observed in the lungs of these animals (Fig 2D). Fig 2E shows a representative photograph highlighting the GFP-labelled EC-Bcl-2 cells along with the metastatic nodule in the lung.

### Endothelial cells expressing Bcl-2 cells show significantly higher E-selectin expression and enhanced tumor cell binding

**Fig 3:**
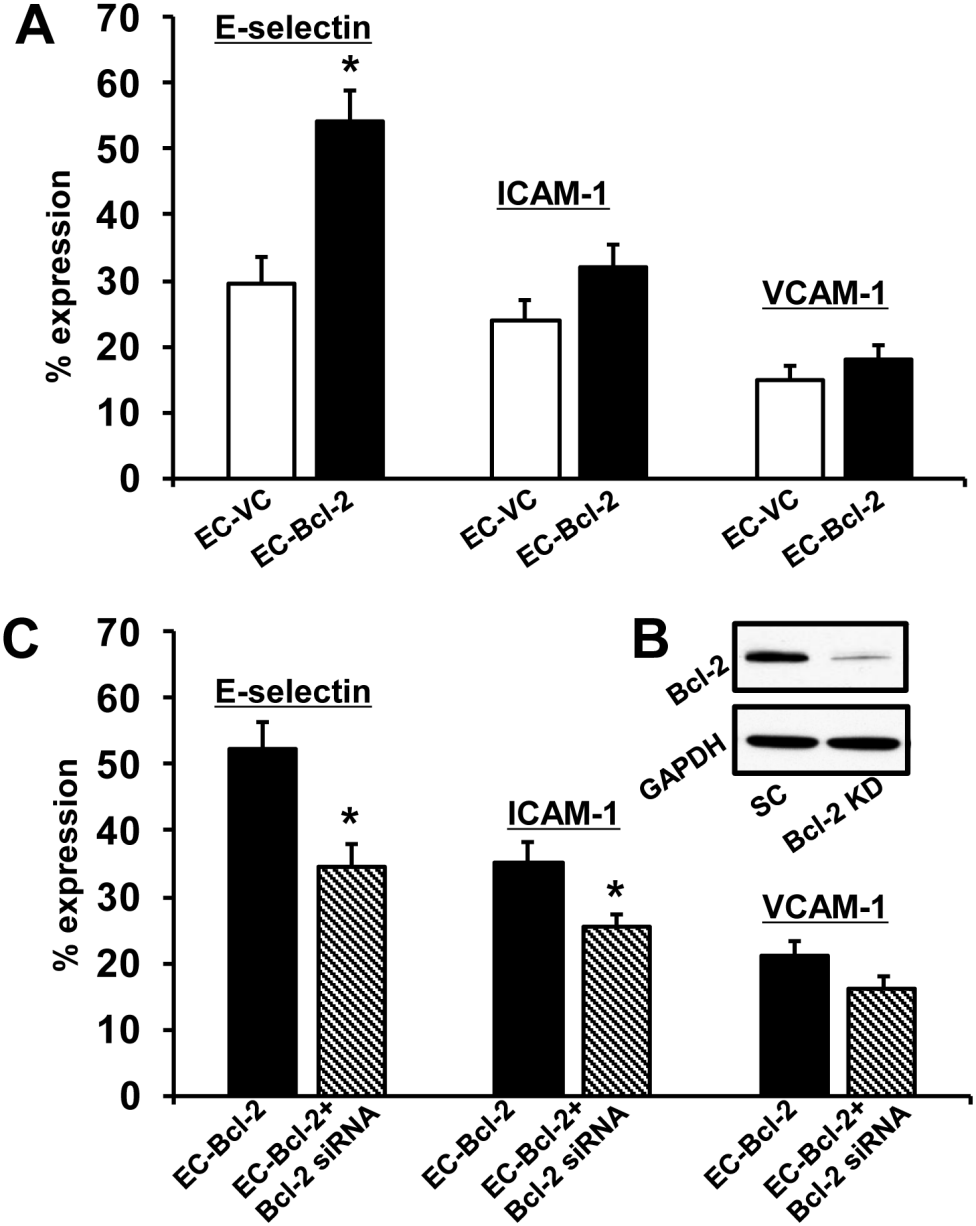
Bcl-2 upregulates adhesion molecules expression. **A;** Adhesion molecule expression on endothelial cells expressing Bcl-2 (EC-Bcl-2) or vector control (EC-VC) was examined by flow cytometry. **B;** siRNA was used to knocked down Bcl-2 expression in EC-Bcl-2 cells. Equal protein loading was verified by stripping the blots and reprobing with tubulin antibody **C;** Adhesion molecule expression on EC-Bcl-2 cells or Bcl-2 knock-down (KD) cells was examined by flow cytometry. The results are expressed as % positive cells ± S.E. *p<0.05.

**Fig 4:**
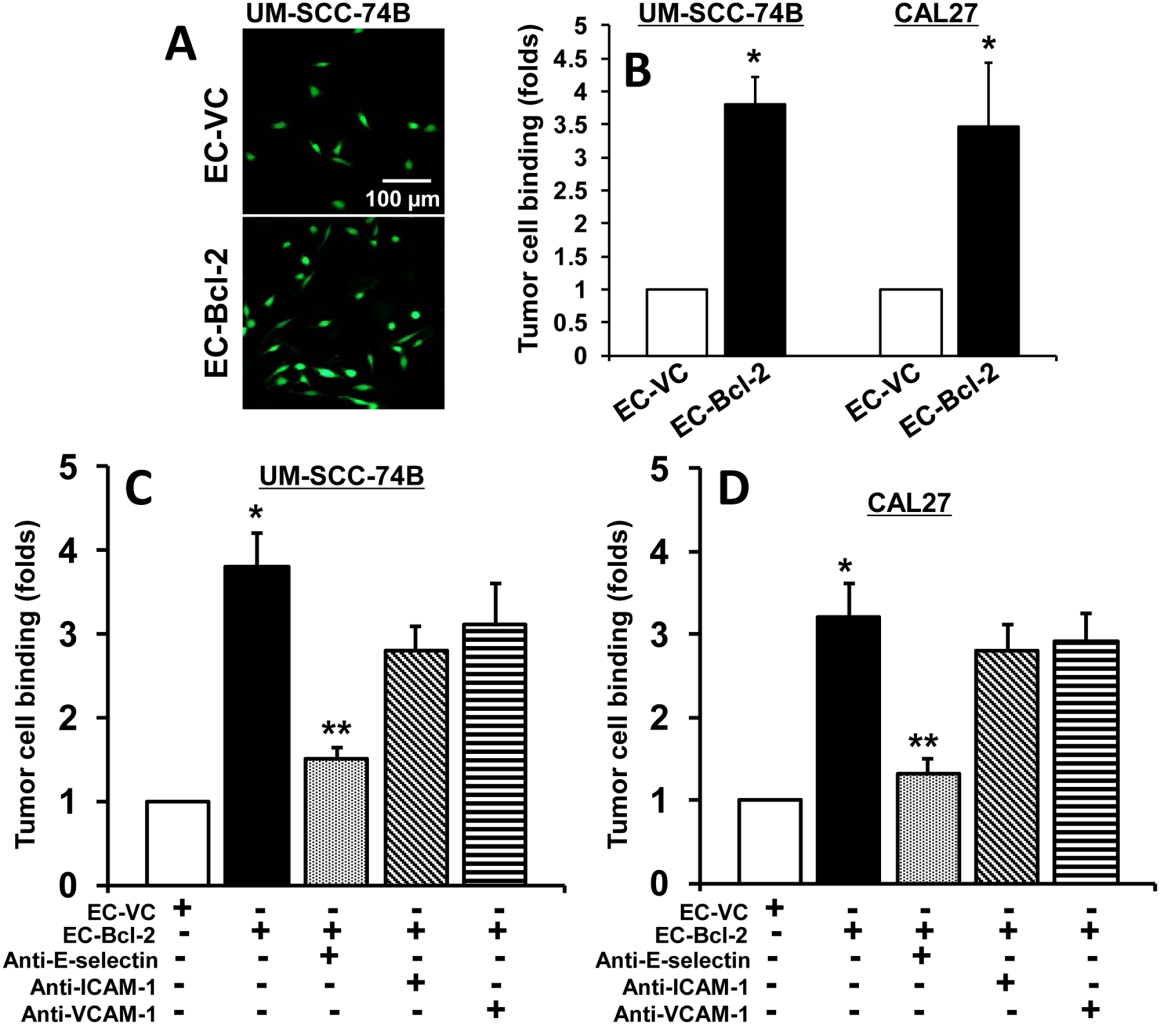
Endothelial cells expressing Bcl-2 (EC-Bcl-2) show enhanced tumor cell binding. **A-B;** EC-Bcl-2 or EC-VC cells were cultured in 8-well chamber slides to form a uniform mono-layer. Tumor cells (CAL27 and UM-SCC-74B) were then pre-labeled with fluorescent dye (calcein-acetoxymethyl ester) and then added to endothelial cells and incubated for an additional 3 hours. At the end of incubation, cells were gently washed and photographed using Nikon Eclipse 80i microscope with DS-Ril camera. The scale bar represents 100 μm. **C-D;** tumor cell binding assays were performed in the presence or absence of neutralizing E-selectin, ICAM-1 or VCAM-1 antibodies. *, represent a significant increase and **, represent a significant decrease in adhesion molecule expression (p<0.05).

We next investigated the effect of Bcl-2 on the expression of adhesion molecule(s) on endothelial cells (ECs). Our results show a significantly higher expression of E-selectin in endothelial cells expressing Bcl-2 (EC-Bcl-2) as compared to vector control cells (EC-VC), whereas we did not observe any significant change in ICAM-1 and VCAM-1 expression (Fig 3A). We next examined if Bcl-2 knockdown in EC-Bcl-2 cells reversed back adhesion molecule(s) expression. Indeed, Bcl-2 knockdown (Fig 3B) significantly decreased adhesion molecule expression in EC-Bcl-2 cells (Fig 3C). In the next set of experiments, we examined if the increased expression of adhesion molecules on endothelial cells lead to enhanced tumor cell binding. Endothelial cells overexpressing Bcl-2 showed (EC-Bcl-2) significantly higher binding to tumor cells (CAL27 and UM-SCC-74B) as compared to vector control cells (Fig 4A and 4B). We next performed tumor cell binding assays in the presence of neutralizing antibodies against E-selectin, ICAM-1 or VCAM-1 to investigate the role these adhesion molecules in tumor cell binding. Anti-E-selectin antibody was able to significantly block tumor cell binding (Fig 4C and 4D), whereas neutralization of ICAM-1 and VCAM-1 did not significantly affect tumor cell binding (Fig 4C and 4D).

### Tumor cells co-cultured with EC-Bcl-2 in non-adherent condition showed significantly higher anoikis resistance

**Fig 5:**
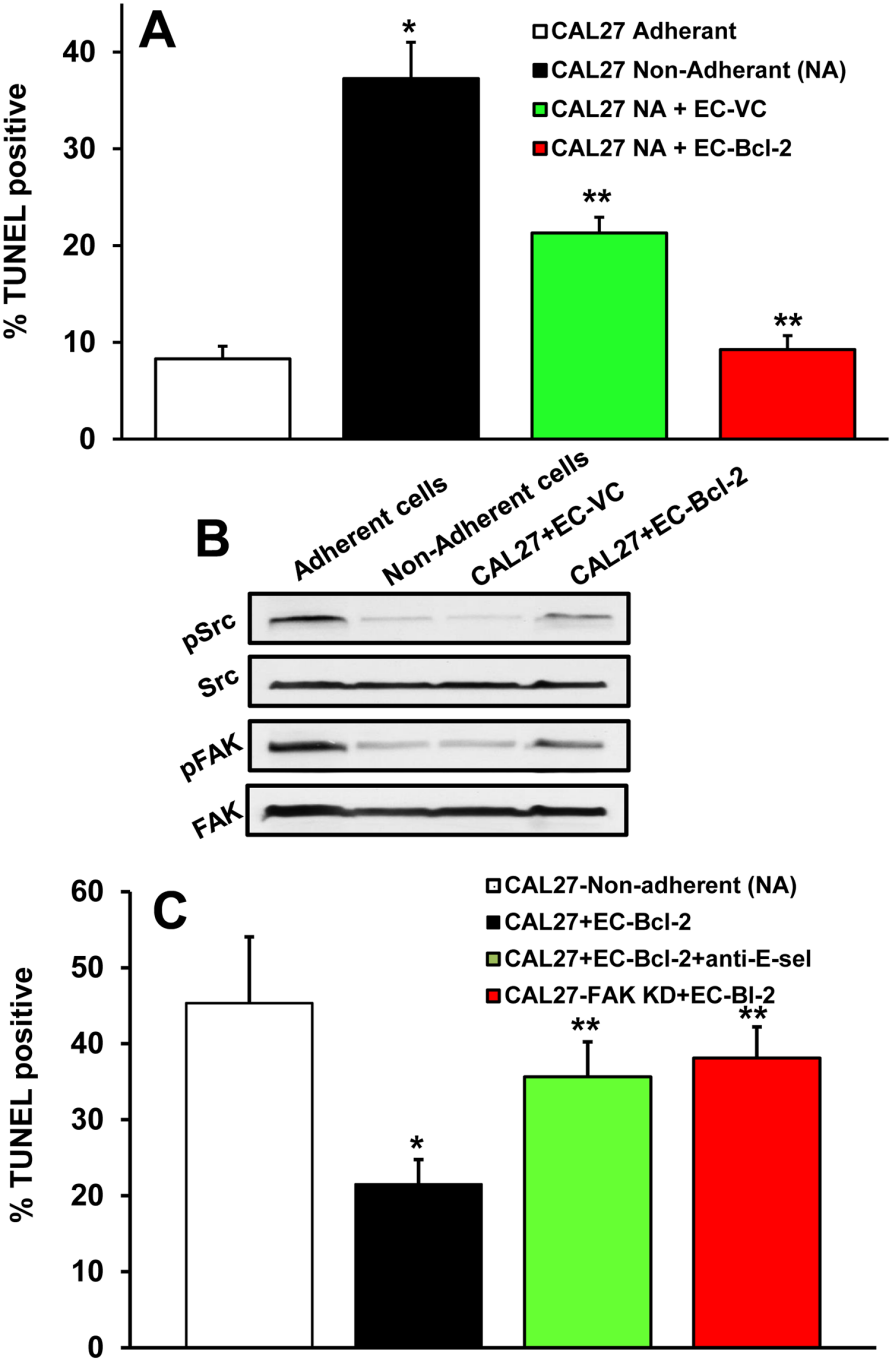
Endothelial cells expressing (EC-Bcl-2) protect tumor cells from anoikis via the Src-FAK signaling pathway. **A-C;** CAL27 cells were co-cultured along with EC-VC or EC-Bcl-2 cells in a non-adherent 6 well plates for 8 hours. At the end of incubation, cells were carefully retrieved and analyzed for anoikis by TUNEL staining **(A)** or cell lysate was prepared and Western blotted for pSrc, pFAK, Src and FAK **(B)**. **C;** tumor cell anoikis assay was performed by either adding neutralizing anti-E-selectin antibody or knocking down FAK in tumor cells.

In this experiment, we examined if endothelial cells expressing Bcl-2 could protect tumor cell from anoikis. CAL27 cells, when cultured alone in non-adherent conditions, showed significantly higher anoikis as compared to CAL27 cells grown in adherent conditions (Fig 5A). In contrast, CAL27 cells co-cultured with EC-Bcl-2 showed a significant decrease in anoikis in non-adherent conditions as compared to CAL27 cells cultured alone. We have previously shown that E-Selectin can activate a number of signaling molecules including Src, PI3K and MAPK [23, 24]. Our results from this study suggest that Src and FAK kinases are activated in tumor cells bound to EC-Bcl-2 in non-adherent conditions (Fig 5B). We next examined if blocking of E-selectin mediated tumor cell binding or knockdown of FAK could reverse EC-Bcl-2 mediated tumor cell anoikis resistance. Indeed, blocking of E-selectin and knockdown of FAK significantly enhanced tumor cell anoikis (Fig 5C).

### Tumor cells co-injected with EC-Bcl-2 via the tail vein showed significantly higher lung metastasis as compared to tumor cells co-injected with control endothelial cells

**Fig 6:**
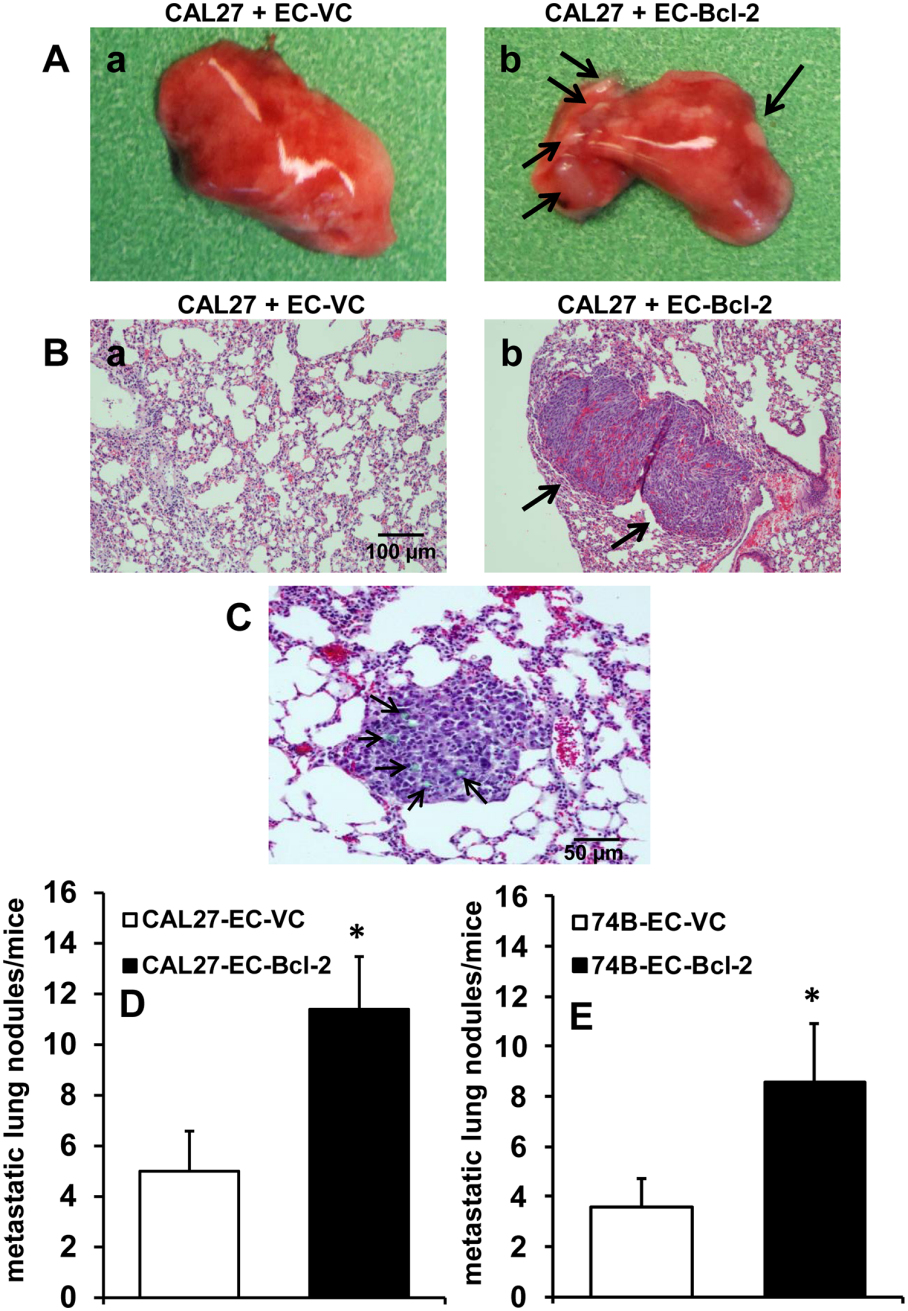
Endothelial cells expressing Bcl-2 (EC-Bcl-2) enhances tumor metastasis by chaperoning tumor cells to lungs. Tumor cells (UM-SCC-74B or CAL27) along with GFP-labeled EC-VC or EC-Bcl-2 were injected into the tail veins of SCID mice. After 3 weeks, tumor metastasis to lungs was analyzed. **A;** Representative images of lungs at day 21. **B;** Histological evaluation of lungs in the same animals at day 21. The scale bar represents 100 μm. **C;** A representative picture showing GFP-labeled ECs (black arrows) along with tumor nodules in lung. The scale bar represents 50 μm. **D-E;** the number of metastatic nodules in the lungs were counted under microscope. *, represents a significant difference (p<0.05).

We used tail vein metastasis model to investigate if tumor cells binding to endothelial cells could protect them in circulation leading to enhanced metastasis to lungs. EC-Bcl-2 or EC-VC (1x10^5^), mixed with tumor cells (UM-SCC-74B or CAL27, 1x10^5^) were injected via the tail vein. After 21 days, lungs were retrieved and the presence of metastatic nodules was analyzed. Animals that were co-injected with tumor cells and EC-Bcl-2 showed significantly higher lung metastasis as compared to tumor cells co-injected with tumor cells and EC-VC (Fig 6). Immunohistochemical analysis of lung sections showed presence of EC-Bcl-2 (GFP-labelled) along with the metastatic nodules in the lungs (Fig 6C). These *in vivo* results further validated our *in vitro* results that binding of tumor cells to endothelial cells protect the tumor cells in circulation and chaperone them to distal metastatic sites.

### EC-Bcl-2 protects tumor cells in non-adhering conditions by mediating a Src-FAK signaling cascade

**Fig 7:**
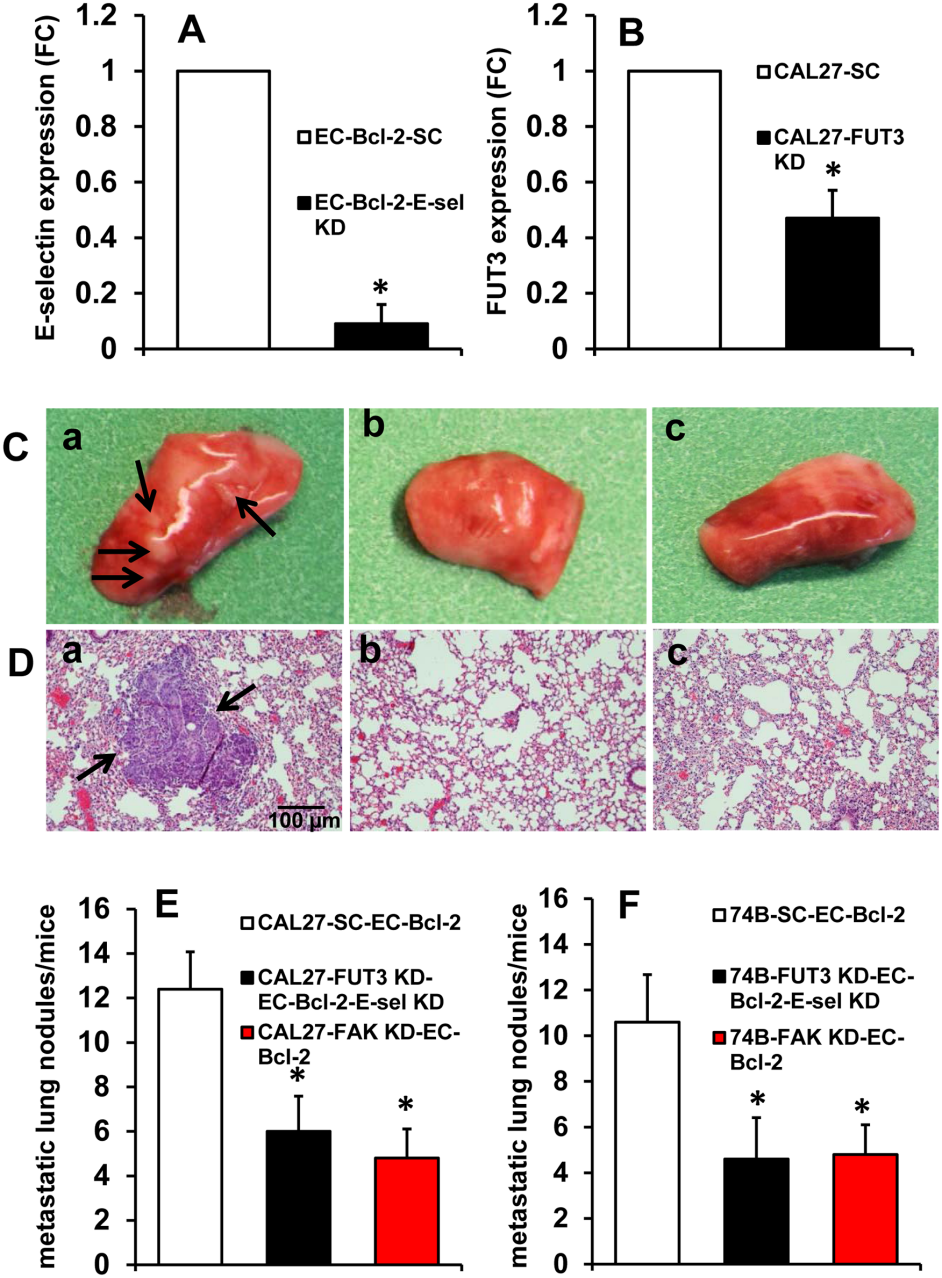
E-selectin knockdown in EC-Bcl-2 or FAK/FUT3 knockdown in tumor cells reverses EC-Bcl-2-mediated tumor metastasis. **A;** E-selectin expression was knocked down in EC-Bcl-2 cells by siRNA. **B;** FUT3 and FAK expression was knocked down in tumor cells (UM-SCC-74B or CAL27) by siRNA. EC-Bcl-2 transduced with scrambled control siRNA (EC-Bcl-2-SC) or tumor cells transduced with scrambled control siRNA (CAL27-SC) were used as control. **C-F;** Tumor cells along with EC-Bcl-2 cells were co-injected into the tail veins of SCID mice. **C;** Representative images of lungs from animals injected with **(a)** CAL27 and EC-Bcl-2 cells, **(b)** CAL27-FUT3 knockdown and EC-Bcl-2- E-selectin knockdown cells and **(c)** CAL27- FAK knockdown and EC-Bcl-2 cells. **D;** Histological evaluation of lungs from animals injected with **(a)** CAL27 and EC-Bcl-2 cells, **(b)** CAL27-FUT3 knockdown and EC-Bcl-2- E-selectin knockdown cells and **(c)** CAL27- FAK knockdown and EC-Bcl-2 cells. Metastatic nodules in lungs are highlighted by black arrows. The scale bar represents 100 μm. **E-F;** the number of metastatic nodules in the lungs were counted under microscope. *, represents a significant difference (p<0.05).

Our *in vitro* experiments suggested that tumor cells may be protected from undergoing anoikis by binding to EC-Bcl-2 through E-selectin and the activation of Src-FAK survival signaling pathway. E-selectin present on the surface of endothelial cells binds to tumor cells predominantly through Sialyl Lewis^x^ or Sialyl Lewis^a^ ligands [25, 26]. The fucose modules of these motifs are essential for their binding function [27]. Fucosylation is catalyzed by fucosyl-transferase enzymes or FUTs. Recently, FUT3 knockdown was shown to disrupt binding of circulating cancer cells to endothelial cells without affecting hematopoietic cell adhesion [28]. We therefore examined the role of E-selection/Sialyl Lewis-mediated binding and its role in the protection of tumor cell from anoikis by knocking down E-selectin in EC-Bcl-2 cells and FUT3 in tumor cells. These cells were co-injected via the tail vein in the SCID mouse model. E-selectin knockdown in EC-Bcl-2 cells and FUT3 knockdown in tumor cells significantly decreased lung metastasis as compared to animals that were co-injected with tumor cells transduced with scrambled siRNA and EC-Bcl-2 (Fig 7).

We next examined the role of FAK in EC-Bcl-2 mediated protection of tumor cells by knocking down FAK in tumor cells and co-injecting them with EC-Bcl-2 via tail vein in the SCID mouse model. FAK knockdown in tumor cells significantly decreased lung metastasis as compared to animals that were co-injected with tumor cells transduced with scrambled siRNA and EC-Bcl-2 (Fig 7).

## Discussion

The metastatic process is a complex multistep process and all the steps must be completed successfully to form distal secondary tumors [29]. The initial steps of this metastatic process that results in the release of tumor cells into microvasculature are relatively more efficient than the survival of these tumor cells in circulation and extravasation into the distal organs [30, 31]. This could be because most of the cancer cells, particularly squamous cancer cells, fail to survive in the harsh anchorage independent conditions of circulation [32] and undergo rapid anoikis [33, 34]. Although our understanding of the molecular and biological events that contribute to the first phase of metastatic process that results in the tumor cell release has increased considerably over the last decade [35], we still know very little about the second phase of tumor metastatic process that includes survival of tumor cells in circulation and development of metastatic lesion at the distal site. In this study, we demonstrate that in addition to tumor cells, tumor-associated endothelial cells are also released from the primary tumor into the circulation. Our results further suggest that activated endothelial cells that express high levels of adhesion molecules could bind to tumor cells, protect them in circulation and chaperone these tumor cells to distal sites. To our knowledge, this is the first report demonstrating a chaperone function for the tumor-associated endothelial cells.

There is extensive data to support the presence of circulating endothelial cells (CECs) in blood samples of cancer patients including head and neck cancer patients [21, 22, 36, 37]. However, it is not completely clear what is the source of these CECs and what role they serve in the cancer patients. In this study, we have demonstrated the presence of Bcl-2 positive CECs in the blood samples from head and neck cancer patients. We think that these Bcl-2 positive CECs are originating from the tumor blood vessels and these could be the activated CECs that Mancuso et al have shown in their study [22]. We have previously shown that tumors from patients with head and neck squamous cell carcinoma exhibit significantly higher expression of Bcl-2 in tumor-associated endothelial cells [6, 9]. However, it is difficult to know with certainty the origin of CECs in the patient blood samples as there are no definitive markers available to distinguish the CECs based on their site of origin. In order to address this question, we stably overexpressed Bcl-2 in human endothelial cells and labeled them with GFP marker. These EC-Bcl-2 cells were then co-implanted with tumor cells in the floor of mouth of SCID mice. Interestingly, blood samples obtained from the animals co-implanted with tumor cells and EC-Bcl-2 showed significantly higher CTCs and CECs as compared to animals co-implanted with tumor cells along with EC-VC. We and others have previously shown that donor human endothelial cells when implanted in SCID mice form capillary network that ultimately links with the murine vasculature to form functional blood vessels within 7 days [6, 38, 39]. In our previous study, we had observed that tumors growth and tumor metastasis to lungs was significantly higher in animals where tumor cells were co-implanted with EC-Bcl-2 as compared to EC-VC. However, EC-Bcl-2-mediated tumor metastasis to lungs was independent of primary tumor size and predominantly mediated by enhanced tumor angiogenesis, blood vessel permeability and tumor cell release [6]. In this study, we also observed that significantly higher CECs are bound to CTCs and co-migrate along with tumor cells to lungs in animals co-implanted with EC-Bcl-2 and tumor cells as compared to EC-VC. These results support our hypothesis that CECs expressing Bcl-2 (CEC-Bcl-2) in the patient blood samples might be originating from tumor microvasculature and they play a role in chaperoning tumor cells to distal sites.

Recent studies have shown that cytokine activated endothelial cells show increased expression of adhesion molecules, particularly E-selectin and this enhanced E-selectin expression was predominantly mediated via the Ras/Raf/MAPK signaling pathway [40, 41]. These studies support our results that Bcl-2 could be mediating enhanced E-selectin expression on endothelial cells by binding to a chaperone protein BAG-1 and activating Raf/MAPK signaling pathway [9, 42]. We further observed in this study that endothelial cells expressing Bcl-2 showed significantly higher tumor cell binding and this endothelial-tumor cell binding was predominantly mediated via the E-selectin. This could be due to the fact that head and neck cancer cells express high levels of E-selectin ligand sialyl lewis-x [43, 44]. Interestingly, high levels of sialyl lewis-x expression was also observed on circulating tumor cells and it was regulated by cancer stem cell (CSC) generation/maintenance pathways [45]. Our results further demonstrate that CECs-Bcl-2, by binding to tumor cell, protect them in non-adherent conditions by providing a temporary substratum. Wong and co-workers have shown that tumor cells can survive and grow intravascular when adherent to endothelial cells in distal capillary beds [46]. Tumor cells have also been shown to acquire increased capacity to survive via the E-selectin mediated PI3K signaling pathway [47]. We have previously shown that E-selectin can activate Src and PI3K signaling pathways [23, 24]. In this study, we observed that binding of tumor cells to endothelial cells induces a marked increase in Src and FAK activation in tumor cells. FAK knockdown significantly reversed EC-Bcl-2 mediated anoikis resistance, *in vitro* and tumor metastasis *in vivo*. A number of studies have demonstrated the role of Src and FAK signaling pathways in cell survival particularly anchorage independent survival [16, 48, 49]. Src activation in cancer cells can lead to phosphorylation of FAK on tyrosine 397 thereby allowing PI3K recruitment [50]. This in turn can activate Akt that inhibits apoptosis by regulating various components of cell death machinery, including Bim [51]. Src-mediated activation of FAK also enhances BAD phosphorylation by Akt and inhibition of caspases (2, 3, 8 and 9), thereby suppressing anoikis [50].

### Conclusions

In this study, we demonstrate a novel function for circulating endothelial cells of providing a temporary substratum to circulating tumor cells in circulation and chaperone these tumor cells to distal sites.

## Materials and Methods

### Cell culture and reagents

Primary human dermal microvascular endothelial cells were purchased from Lonza (Allendale, NJ) and were cultured in EGM-2 medium supplemented with growth factors. Head and neck squamous carcinoma (HNSCC) cell line CAL27 was obtained from ATCC and UM-SCC-74B was obtained from Dr. Thomas Carey (University of Michigan) [52]. The identity of all the cancer cell lines was confirmed by STR genotyping (Identifiler Kit, Applied Biosystems, Carlsband, CA). HNSCC cell lines were cultured in DMEM supplemented with 10% fetal bovine serum (FBS) containing 1% penicillin/streptomycin and 1% non-essential amino acids. Antibodies against E-selectin (clone 68-5H11), ICAM-1 (clone HA58) and VCAM-1 (clone 51-10C9) for flow cytometry were purchased from BD Biosciences (San Jose, CA). Neutralizing antibodies against E-selectin (clone BBIG-E6), ICAM-1 (clone BBIG-11) and VCAM-1 (clone BBIG-V1) were purchased from R&D Systems (Minneapolis, MN). Primary antibody against pFAK (Y^397^, ab4803) was obtained from Abcam (Cambridge, MA). Mouse monoclonal anti-human CD31 antibody (JC70A) was obtained from Dako (Glostrup, Denmark). pSrc (Y^416^, catalog # 2101), Src (catalog # 2108), FAK (catalog # 3285) and tubulin (catalog #2148) antibodies were purchased from Cell Signaling (Danvers, MA). APO-BRDU terminal deoxynucleotidyl transferase (TdT)-mediated dUTP-biotin nick end labeling (TUNEL) assay kit was purchased from Sigma (St. Louis, MO). Calcein-acetoxymethyl ester dye was purchased from Molecular Probes (Eugene, OR). Transfection and Western blotting reagents were purchased from Invitrogen (Carlsbad, CA).

### Patient samples and circulating endothelial cell (CEC) characterization

Peripheral blood samples were collected from head and neck cancer patients visiting the head and neck oncology clinic at the University of Michigan Cancer Center prior to surgery or starting a new therapeutic treatment. Use of patient samples was approved by the University of Michigan Institutional Review Board, and written informed consent was obtained from all participants. Normal blood samples were collected from healthy volunteers. CEC isolation was performed according to previously reported methods [21, 53]. In brief, peripheral blood samples were collected in green-top BD Vacutainer blood collection tubes containing sodium heparin. Blood samples were then mixed with RBC lysis buffer (1:10, eBioscience, San Diego, CA) and incubated for 10 min at room temperature (RT). The reaction was stopped by adding 20 ml of PBS and cells were collected by spinning them at 300g for 10 min. The cells were washed, counted and resuspended in CEC isolation buffer (PBS containing 2% FBS and 1mM EDTA) at 2x10^8^ cells/ml. To block non-specific binding, 100 μl/ml of FcR blocking antibody was added. To label the endothelial cell lineage cells, anti-CD146-PE antibody (1μg/ml) was added and incubated for 15 min at RT. The cells were washed twice and further incubated with anti-mouse PE antibody. The PE labeled cells were then isolated using Easy Sep PE selection cocktail containing magnetic nanoparticles (Stem Cell Technologies). The unbound cells are removed by magnetic separation. PE-labeled bound cells were counted and then cytospun on glass slides. CECs were further stained for factor VIII (von Willibrand factor) or Bcl-2 using DAB system (EnVision+, DakoCytomation).

### Retroviral vector construction and HDMEC transduction

Bcl-2 and green fluorescence protein (GFP) were stably overexpressed in endothelial cells using retroviral vector as described previously [54]. The Bcl-2, GFP or vector alone constructs were introduced into PA317 amphotropic packing cells with Lipofectamine (Life Technology, Grand Island, NY). Viral supernatants were collected after 24 hours, centrifuged, filtered, and stored at -70°C. ECs were transduced with Bcl-2, GFP or control vector by overnight incubation with viral supernatant in the presence of 4 μg/ml polybrene. Endothelial cell growth medium supplemented with 400 μg/ml G418 was used to select the resistant clones. Bcl-2 expression was confirmed by Northern and Western blot analysis.

### Transient transfection with siRNA

siGENOME SMART pool siRNAs from Dharmacon were used to knockdown Bcl-2 and E-selectin in endothelial cells and FAK and FUT3 in tumor cells according to the manufacturer’s instructions. Seventy two hours post transfection, Bcl-2, E-selectin, FAK and FUT3 knockdown was verified by RT-PCR or Western blotting and cells were used for adhesion molecule expression, tumor cell binding or tumor cell anoikis experiments.

### Analysis of adhesion molecule expression

Adhesion molecule(s) expression of E-selectin, ICAM-1 and VCAM-1 on endothelial cells was measured by flow cytometry. Endothelial cells expressing Bcl-2 (EC-Bcl-2) or VC (EC-VC) were cultured in 10 cm dishes. Cells were harvested from the petri dishes in ice-cold PBS-EDTA (5mM) medium using cell scrapper. Cells were washed with wash buffer (PBS + 0.1% sodium azide + 1% FBS), resuspended in 50 μl of wash buffer and incubated with primary antibodies (E-selectin, ICAM-1 or VCAM-1; 1:100 dilutions) for 30 min at RT. At the end of incubation, cells were washed and further incubated with goat anti-mouse FITC antibody (1:100, Sigma) for 30 min at RT. Cells were washed twice and fixed (BD cytofix, BD Biosciences) and analyzed with flow cytometry. Ten thousand cells from each sample were analyzed.

### Tumor cell binding assay

EC-Bcl-2 or EC-VC cells were plated in 6-well plates and incubated until they formed a uniform monolayer. Tumor cells (CAL27 or UM-SCC-74B) were pre-labeled with a fluorescent dye by incubating them with 5 μM calcein-acetoxymethyl ester (calcAM, Molecular Probes, Eugene, Oregon) for 30 min at 37°C. Labeled tumor cells were then added to endothelial cell monolayer and incubated for 3 hour at 37°C. At the end of incubation, cells were gently washed 3 times and the binding of tumor cells to endothelial cells was analyzed using a fluorescent microscope (Nikon Eclipse 80i, Nikon, Melville, NY). The numbers of tumor cell bound were quantified in 10 fields in each well.

### TUNEL assay

To evaluate the tumor cell anoikis in non-adherent conditions, tumor cells (5 × 10^5^) were cultured in non-adherent 6-well plates (Corning) for 8 hours. Tumor cells cultured in adherent conditions were used as a control. At the end of incubation, cells were carefully retrieved and analyzed by TUNEL staining for anoikis [12]. In brief, tumor cells were fixed with cytofix buffer (BD cytofix, BD Biosciences, San Jose, CA) for 15 minutes at 4°C, and then stored overnight in 70% ethanol at -20°C. The percentage of apoptotic cells were then evaluated using the APO-BRDU terminal deoxynucleotidyl transferase (TdT)-mediated dUTP-biotin nick end labeling (TUNEL) assay according to the manufacturer’s instructions (Sigma, St. Louis, MO). Apoptotic tumor cells were quantitated by flow cytometry using an argon laser excited at 488 nm (BD Biosciences, San Jose, CA).

### Western blot analysis

Whole cell lysates were separated by 4–12% NuPAGE Bis-Tris gels (Invitrogen, Carlsbad, CA) and transferred onto PVDF membranes. Nonspecific binding was blocked by incubating the blots with 3% BSA in Tris buffered saline containing 0.1% Tween-20 (TBST) for 1 hour at room temperature (RT). The blots were then incubated with primary antibody in TBST + 3% BSA at 4°C overnight. After washing with TBST, the blots were incubated with horseradish peroxidase-conjugated sheep anti-mouse IgG (1:10,000) or with donkey anti-rabbit IgG (1:10,000) for 1 hour at RT. An ECL-plus detection system (Amersham Life Sciences, Piscataway, NJ) was used to detect specific protein bands. Protein loading in all the experiments was normalized by stripping the blots and then re-probing with anti-tubulin antibody.

### Orthotopic head and neck cancer model

All animal work has been conducted according to the Ohio State University IACUC Animal ethic committee and was approved by this committee (Animal Welfare Assurance Number A3261-01). Tumor cells (1 x 10^6^) and GFP-labeled EC-Bcl-2 or EC-VC cells (1 x 10^6^) were mixed with Matrigel and 50 μl of this mixture is carefully injected into the floor of mouth of anesthetized (Ketamine/xylazine cocktail) SCID mice. Tumor burden in the animals was monitored daily and it was not allowed to exceed the 10% of the animal’s weight. After 14 days, blood samples were collected. At the end of study, animals were sacrificed by CO_2_ asphyxiation and lungs were carefully removed and analyzed for the presence of GFP-labeled CECs and CTCs by immunohistochemistry.

### *In vivo* tumor metastasis assay

SCID mice ranging in weight from 18 to 25 gm were used for this study. Tumor cells (1x10^5^) along with endothelial cells expressing Bcl-2 (EC-Bcl-2, 1x10^5^) or vector alone (EC-VC) were co-injected in the SCID mice via tail vein using 30 gauge needles. After 3 weeks, lungs were carefully removed, fixed with 4% paraformaldehyde and then processed for immunohistochemistry. Paraffin embedded slides were stained for hematoxylin and eosin (H&E) and presence of metastatic nodules in lungs was analyzed and photographed using Nikon Eclipse 80i microscope with DS-Ri1 camera.

### Statistical analysis

Data from all the experiments are expressed as mean ± SEM. Statistical differences were determined by two-way analysis of variance and Student’s t test. A p value of <0.05 was considered significant.
